# Impact of Frailty on Early Readmissions of Endoscopic Retrograde Cholangiopancreatography in the United States: Where Do We Stand?

**DOI:** 10.3390/jcm13206236

**Published:** 2024-10-18

**Authors:** Bhanu Siva Mohan Pinnam, Dushyant Singh Dahiya, Saurabh Chandan, Manesh Kumar Gangwani, Hassam Ali, Sahib Singh, Umar Hayat, Amna Iqbal, Saqr Alsakarneh, Fouad Jaber, Islam Mohamed, Amir Humza Sohail, Neil Sharma

**Affiliations:** 1 Department of Internal Medicine, John H. Stroger, Jr. Hospital of Cook County, Chicago, IL 60612, USA; 2 Division of Gastroenterology, Hepatology & Motility, The University of Kansas School of Medicine, Kansas City, KS 66160, USA; 3 Division of Gastroenterology and Hepatology, Creighton University School of Medicine, Omaha, NE 68178, USA; 4 Department of Gastroenterology and Hepatology, University of Arkansas for Medical Sciences, Little Rock, AR 72205, USA; 5 Division of Gastroenterology, Hepatology & Nutrition, East Carolina University, Brody School of Medicine, Greenville, NC 27834, USA; 6 Department of Internal Medicine, Sinai Hospital, Baltimore, MD 21215, USA; 7 Department of Internal Medicine, Geisinger Wyoming Valley Medical Center, Wilkes Barre, PA 18711, USA; 8 Department of Internal Medicine, University of Toledo Medical Center, Toledo, OH 43614, USA; 9 Department of Internal Medicine, University of Missouri-Kansas City, Kansas City, MO 64110, USA; 10 Division of Gastroenterology and Hepatology, Baylor College of Medicine, Houston, TX 77030, USA; 11 Complex Surgical Oncology, Department of Surgery, University of New Mexico, Albuquerque, NM 87131, USA; 12 Peak Gastroenterology, Gastrocare Partners, UCHealth, Colorado Springs, CO 80920, USA

**Keywords:** frailty, endoscopic retrograde cholangiopancreatography, readmissions, outcomes, mortality

## Abstract

**Background/Objectives:** We assessed the impact of frailty on outcomes of endoscopic retrograde cholangiopancreatography (ERCP) in the United States. **Methods:** The National Readmission Database (2016–2020) was used to identify index and 30-day ERCP readmissions, which were categorized into low-frailty, intermediate-frailty, and high-frailty groups based on the Hospital Frailty Risk Score (HFRS). Outcomes were then compared. **Results:** Of 885,416 index admissions, 9.9% were readmitted within 30 days of ERCP. The odds of 30-day readmission were higher in the intermediate-frailty group (12.59% vs. 8.2%, odds ratio [OR] 1.67, 95% confidence interval [CI] 1.64–1.71, *p* < 0.001) and the high-frailty group (10.57% vs. 8.2%, OR 1.62, 95% CI 1.52–1.73, *p* < 0.001) compared to the low-frailty group. On readmission, a higher HFRS also increased mean length of stay (intermediate-frailty vs. low-frailty: 8.49 vs. 4.22 days, mean difference (MD) 4.26, 95% CI 4.19–4.34, *p* < 0.001; high-frailty vs. low-frailty: 10.9 vs. 4.22 days, MD 10.9 days, 95% CI 10.52–11.28, *p* < 0.001) and mean total hospitalization charges (intermediate-frailty vs. low-frailty: $118,996 vs. $68,034, MD $50,962, 95% CI 48, 854–53,069, *p* < 0.001; high-frailty vs. low-frailty: $195,584 vs. $68,034, MD $127,550, 95% CI 120,581–134,519, *p* < 0.001). The odds of inpatient mortality were also higher for the intermediate-frailty and high-frailty compared to the low-frailty subgroup. **Conclusions:** Frailty was associated with worse clinical outcomes after ERCP.

## 1. Introduction

Frailty, defined as an aging-related syndrome of physiological decline, is characterized by diminished ability to cope with acute stressors. Numerous physical and psychosocial determinants are factored in when defining frailty. Patient frailty has been identified as an independent risk factor for adverse clinical outcomes for a wide array of medical conditions and procedures [1,2,3,4,5,6]. Furthermore, studies have demonstrated that frailty increases healthcare resource utilization and adds to the economic burden on the healthcare sector [7]. As an example, a UK-based longitudinal study estimated that frailty accounts for an added £5.8 billion/year of healthcare-related expenditure across the country [8]. Despite its ubiquity and significant impact on healthcare outcomes, quantifying frailty has been rather challenging. The lack of such quantification has continued to be a significant barrier to the incorporation of frailty into medical decision-making and physician stewardship of healthcare resources. Furthermore, in addition to being inaccurate, previously used frailty assessment tools were often criticized for being quite cumbersome. These tools required varying degrees of manual assessment, which led to a lack of objectivity.

Developed by Gilbert et al. using the International Classification of Diseases (ICD) codes, the hospital frailty risk score (HFRS) has revolutionized assessment of frailty, particularly in an inpatient setting [9]. Using a set of 109 ICD-10 codes that are highly prevalent in frail patients, Gilbert et al. produced a model to weigh the impact of each diagnosis code on frailty. Essentially, an individual’s HFRS is the arithmetic sum of these numeric values. Using this score, the spectrum of frailty can be categorized, making a comparison of individuals in each category possible [9,10,11,12].

Endoscopic retrograde cholangiopancreatography (ERCP), a vital procedure in the arsenal of therapeutic endoscopists, involves the utilization of a side-viewing endoscope under fluoroscopic guidance to perform a wide variety of interventions for pancreaticobiliary disorders [13]. Due to its widespread availability and minimally invasive nature, the utilization of ERCP is on the rise [14]. Advancements in endoscopic techniques and increasing operator experience have led to ensuring the safe use of ERCP for therapeutic purposes [15]. However, there is a significant paucity of data on the impact of frailty in individuals receiving ERCP, particularly in an inpatient setting. Hence, in this study, we aimed to evaluate the utility of the HFRS in predicting outcomes for inpatient use of ERCP. We used one of the largest publicly available healthcare databases, the nationwide readmissions database (NRD), to study the impact of the HFRS on readmission rates, inpatient mortality, and the healthcare burden in the United States (US).

## 2. Materials and Methods

### 2.1. Data Source

The data analyzed for this comparative retrospective study were derived from the NRD. The NRD is the largest, publicly available readmission database in the US maintained by the Agency for Healthcare Research and Quality (AHRQ) Healthcare Cost and Utilization Project (HCUP) State Inpatient Databases [16]. For each calendar year, NRD contains discharge information from geographically dispersed and diverse states. It stores both patient-level and hospital-level information, and the hospitals are stratified according to ownership control, the number of beds, teaching status, and metropolitan/non-metropolitan location, using the International Classification of Disease (ICD) codes. The NRD allows for a weighted analysis to obtain 100% of the US readmissions within a given year.

### 2.2. Study Population

**Figure 1:**
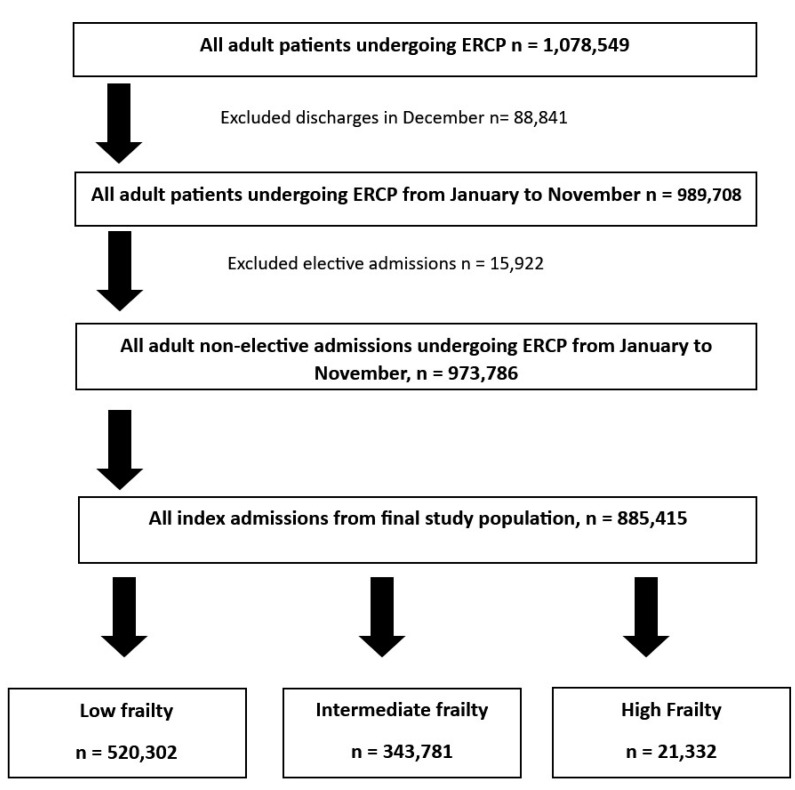
Inclusion and exclusion criteria used to derive the study sample of adult patients undergoing ERCP on index admission.

The NRD was utilized from 2016 to 2020 to identify all adult (≥18 years old) hospitalizations that included therapeutic ERCP during the index hospitalization. Using unique hospitalization identifiers, subsequent hospitalization within 30 days of the index hospitalization were tagged as readmission. Individuals under the age of 18 years, traumatic admissions, and elective hospitalizations were excluded from the analysis (Figure 1).

### 2.3. Hospital Frailty Risk Score

ICD-10 codes listed by Gilbert et al. in the original development and validation model were identified in the index admissions receiving ERCP [9]. The same numeric scoring used by Gilbert et al. was used, and the sum of these scores was used to assign HFRS to each admission. They were then classified into low-, intermediate-, and high-frailty groups if their HFRS scores were <5, 5–15, and >15, respectively. These scores were calculated for a single index admission and not cumulatively, ensuring a more accurate frailty status relevant to the admission.

### 2.4. Statistical Analysis and Outcome Measures

The data were analyzed using Stata^®^ Version 17 software (StataCorp, College Station, TX, USA). All analyses were performed using weighted samples for national estimates in adjunct with HCUP regulations for utilization of the NRD. Hospitalization characteristics were extracted directly from the NRD. The comorbidity burden was quantified using the Charlson Comorbidity Index (CCI) score. The study population was stratified based on gender, age categories, CCI score, insurance type, hospital bed-size, and the odds of 30-day readmission. Mortality within each stratum was calculated using univariate regression analysis with HFRS as the independent variable. Linear regression was used to compare the length of stay (LOS) and total hospitalization charges (THC) at increasing levels of frailty. The THC from 2016 through 2020 was adjusted for inflation in the healthcare sector using the Consumer Price Index (CPI) inflation calculator maintained by the U.S. Bureau of Labor statistics. The Rao–Scott adjusted chi-square test was used in identifying proportions of index and readmission diagnoses.

### 2.5. Ethical Considerations

The NRD lacks patient- and hospital-specific identifiers. Therefore, this study was exempt from Institutional Review Board (IRB) approval as per the guidelines put forth by our institutional IRB for analysis of the NRD database.

## 3. Results

### 3.1. Index Hospitalization Characteristics

Between 2016 and 2020, 885,416 index admissions were identified to have undergone ERCP. Of the index admissions, 520,302 (58.76%) had low frailty, 343,781 (38.83%) had intermediate frailty, and 21,332 (2.41%) had high frailty. As expected, the mean age for low-, intermediate-, and high-frailty index admissions were 55.2, 68.3, and 76.35 years, respectively. The high-frailty group had the highest comorbidity burden with 66.8% of these admissions having a CCI of 3 or more (compared to 16.8% in those with low frailty, and 47.4% in those with intermediate frailty). Furthermore, most of these admissions were largely located in metropolitan areas. Hospitalization characteristics are highlighted in Table 1, Table 2 and Table 3. Indications for ERCP were identified within each cohort. Choledocholithiasis was the most commonly identified indication in all three cohorts (71.8%, 73.49% and 72.64% in the low-, intermediate-, and high-frailty cohorts). This was followed by biliary pancreatitis (28.11%, 25.69%, and 19.59% in the low-, intermediate-, and high-frailty cohorts). A small proportion of the intermediate-frailty (0.13%) and high-frailty (1.21%) admissions underwent ERCP for cholangitis. These distribution of indications for ERCP in the three cohorts is highlighted in Table 4.

### 3.2. 30-Day Readmissions

The overall 30-day readmission rate for patients who underwent ERCP on index admission was noted to be 9.9%. From index admissions, 8.2%, 12.59%, and 10.57% of patients were readmitted within 30 days in the low-, intermediate-, and high-frailty subgroups, respectively. We noted higher odds of 30-day readmission in the intermediate-frailty (12.59% vs. 8.2%, odds ratio [OR] 1.67, 95% confidence interval [CI] 1.64–1.71, *p* < 0.001) and the high-frailty groups (10.57% vs. 8.2%, OR 1.62, 95% CI 1.52–1.73, *p* < 0.001) compared to the low-frailty group (Table 5).

Upon stratifying by gender, although higher HFRS was associated with higher odds of 30-day readmission for both genders, we noted relatively higher odds of readmission for females in the intermediate-frailty (OR 1.9, 95% CI 1.84–1.96, *p* < 0.001) and higher-frailty subgroups (OR 1.87, 95% CI 1.72–0.04, *p* < 0.001) compared to men (OR 1.41, 95% CI 1.37–1.46, *p* < 0.001 with intermediate frailty and OR 1.34, 95% CI 1.23–1.47, *p* < 0.001 with severe frailty).

CCI score significantly impacted the predictive capacity of HFRS. Frailty was associated with higher odds of 30-day readmission in patients with a CCI of 1 (OR 1.53, 95% CI 1.45–1.61, *p* < 0.001 in the intermediate-frailty subgroup and OR 1.49, 95% CI 1.25–1.79, *p* < 0.0.001 in the high-frailty subgroup). Interestingly, in admissions with a CCI score >1, higher frailty did not increase the odds of readmission. Readmission characteristics are highlighted in Table 1, Table 2 and Table 3.

Through multivariate regression analysis, after adjusting for potential confounders and indication for ERCP, we noted higher odds of 30-day readmission in the intermediate-frailty (adjusted odds ratio [aOR] 1.26, 95% CI 1.23–1.26, *p* < 0.001) and the high-frailty groups (aOR 1.12, 95% CI 1.04–1.19, *p* = 0.001) compared to the low-frailty group. These findings are highlighted in Table 6.

### 3.3. Inpatient Mortality

The overall inpatient mortality rates were 1.66% and 4.42% for index and 30-day readmissions of ERCP, respectively. With an increase in frailty, we noted higher inpatient mortality for both index and 30-day readmissions. The 30-day readmissions had a higher mortality rate in the low-frailty (1.11% vs. 0.17%, OR 6.86, 95% CI 5.86–8.03, *p* < 0.001), intermediate-frailty (7.3% vs. 3.49%, OR 2.17, 95% CI 2.04–2.31, *p* < 0.01), and high-frailty subgroups (10.44% vs. 8.2%, OR 1.29, 95% CI 1.04–1.60, *p* < 0.001) compared to index admissions. Inpatient mortality is highlighted in Table 7.

### 3.4. Healthcare Burden

The mean THC was higher in the intermediate-frailty ($118,996 vs. $68,034, MD $50,962, 95% CI 48, 854–53,069, *p* < 0.001) and high-frailty ($195,584 vs. $68,034, MD $127,550, 95% CI 120,581–134,519, *p* < 0.001) subgroups compared to the low-frailty subgroup. Furthermore, we also noted longer mean LOS for the intermediate-frailty (8.49 vs. 4.22 days, MD 4.26, 95% CI 4.19–4.34, *p* < 0.001) and high-frailty (10.9 vs. 4.22 days, MD 10.9 days, 95% CI 10.52–11.28, *p* < 0.001) subgroups compared to the low-frailty subgroup. The healthcare burden is highlighted in Table 8.

### 3.5. Readmission Diagnoses

We evaluated the most commonly identified principal diagnoses on 30-day readmission within each cohort. Sepsis was the most single most commonly identified readmission diagnosis in each cohort; however, the proportion of readmissions for sepsis was much higher in the intermediate- and high-frailty cohorts (14.8% and 22.22%) compared to the low-frailty cohort (7.9%). Other important principal diagnoses on readmission included pancreatic cancer, acute pancreatitis, choledocholithiasis, cholangiocarcinoma, and cholangitis in the low-frailty cohort. These readmission diagnoses have been highlighted in Table 9.

## 4. Discussion

Our study highlights that increasing frailty is associated with an increase in the rate of 30-day readmission in patients undergoing ERCP, ranging from 8.2% to 12.59% based on the degree of frailty. This increase in odds of readmission was higher for females compared to males. Frailty increased readmission rates in all age groups; However, the presence of frailty in younger patients had a more dramatic increase in the odds of 30-day readmissions. Overall, 30-day readmissions had higher odds of mortality compared to index admissions. Furthermore, the THC and LOS increased significantly with higher frailty scores. In clinical practice, quantifying the impact of frailty on outcomes in patients undergoing ERCP is crucial as it allows therapeutic endoscopists to prognosticate such patients and take preemptive measures to reduce the risk of unfavorable outcomes in these high-risk individuals.

Previous studies have established an association between frailty and readmissions rates for numerous pathologies such as chronic pancreatitis, inflammatory bowel disease, heart failure, and many more [17,18,19]. Despite a clinical understanding of the fact that frail individuals have worse clinical outcomes with ERCP, there is limited data on the impact of frailty on ERCP outcomes [20,21,22,23,24]. Furthermore, the extent of frailty’s impact on these outcomes is also poorly understood, owing to the dearth of real-world data, which has made operationalizing frailty quite challenging. A possible solution for this could be the effective use of HFRS as a frailty indicator. A major finding of our study was increased 30-day readmission rates for frail patients. This finding is in line with a previously published study in 2017 that had a smaller sample size and demonstrated that frail patients undergoing ERCP experienced a higher readmission rate [20]. Another 4-year national study also had similar findings to our study as the authors noted that frailty increased the odds of readmission in admissions for acute biliary pancreatitis [21]. Furthermore, frail patients may have multiple readmissions owing to procedural complications, to which they are more prone compared to the general population [25,26,27,28]. Like any other medical intervention, ERCP is not without risks, and frailty has emerged as a potential predictor for worse clinical outcomes following ERCP. Additional large prospective studies are required to examine the impact of frailty on the incidence of post-ERCP complications, most importantly post-ERCP pancreatitis. An interesting finding in our study was that of a relatively higher readmission risk with increasing frailty in younger patients. There is a substantial amount of literature evaluating frailty in the aged. However, there is scarcity of data on frailty in the young, making this a relatively challenging aspect to contextualize. This finding of our study sheds light on the importance of addressing frailty in the younger patient population. We hypothesize that the fundamental difference in the etiological basis of frailty in younger patients and the psychosocial impact of such frailty play a key role in this finding. Further prospective studies are essential to selectively study the impact of frailty on young patients undergoing ERCP.

Interestingly, another key finding of our study was that in admissions with a CCI score >1, higher frailty did not increase the odds of readmission. This meant that with an increasing comorbid burden, readmission rates become somewhat similar between frail and non-frail patients. This finding further strengthens the reliability of HFRS and reveals the true impact of frailty in individuals with a lower comorbidity burden. CCI scores have previously been used extensively as predictors of readmission in admissions for biliary pathologies [29,30]. However, in patients with lower or no comorbidities, anticipating readmissions becomes more challenging, and limited tools exist to predict readmissions in this subset population. Based on our results, HFRS could prove to be an effective tool for therapeutic endoscopists to screen for and anticipate a higher likelihood of readmission in this subset population.

In our study, we observed that frailty is associated with a significantly higher odds of readmission in females than in males. There are likely multiple reasons for this finding. Firstly, gender-based physiological differences are associated with a higher degree of frailty in women. Low muscle mass and post-menopausal changes attribute a higher degree of frailty to women. Additionally, prior studies have shown that women have a higher risk of post-ERCP complications like post-ERCP pancreatitis [31], which further increases the short-term readmission risk. There are also studies showing that polypharmacy is more prevalent in women [32], which further increases the risk of adverse events. Furthermore, frail women, especially elderly women, may have less robust social support than men, which potentially contributes to poorer care post-discharge, which culminates in higher readmission risk. Women also have a higher prevalence of anxiety and depression [33], which is further worsened by the presence of frailty. This is known to limit optimal post-discharge care, further contributing to a higher readmission burden.

In our study, sepsis was the most commonly identified readmission diagnosis in all three cohorts. This is explained by the association of sepsis with multiple indications for which ERCP is undertaken. These include cholangitis, acute pancreatitis, malignant biliary obstruction, choledocholithiasis with incomplete stone clearance, and so forth. Pancreatic cancer and cholangiocarcinoma were also noted to be significant diagnoses on 30-day readmission. Malignant biliary obstruction can prove to be a therapeutic challenge. ERCP is often undertaken as a measure to provide palliation in this patient population. Owing to the advanced stages at which these malignancies are often diagnosed and the poor therapeutic response encountered in such patients, an inherently high readmission risk is often observed. This is evident in our study as well, where these diagnoses contributed to significant readmission burden across varying degrees of frailty.

Higher frailty is associated with reduced physical activity, multi-morbidity, disability, and adverse outcomes [34]. These can lead to an increased healthcare burden and utilization, in terms of LOS, THC, and inpatient mortality, as observed in our study. In prior published literature, frailty has been also linked to worse in-hospital outcomes with exposure to operative stress [35,36]. A prospective cohort study of 200 patients admitted for acute biliary conditions noted that frail individuals experienced higher rates of mortality and incidence of peri-procedural complications, which translated to longer hospital stays and THC, similar to our study [28]. This further demonstrates that frail patients require more complex, thorough, and multi-disciplinary care to optimize health at discharge and reduce the burden of readmissions. Furthermore, screening for frailty and addressing modifiable factors may be an important factor in optimizing outcomes and reducing the risk of adverse events following ERCP. Further research is needed to develop and validate frailty-screening tools specifically for ERCP and to determine the most effective interventions to mitigate the impact of frailty on procedural outcomes. A multifaceted approach to improve frailty in this high-risk population is crucial. In our study, we only included non-elective admissions. Such admissions need ERCP on a more time-sensitive basis, compared to elective admissions. While this could limit the potential measures to improve frailty pre-procedurally, there is still scope to implement post-procedural measures to reduce frailty-related adverse outcomes. Physical and nutritional rehabilitation, psychosocial support, and vocational rehabilitation could be utilized in optimizing periprocedural patient outcomes and in reducing readmission burden following ERCP, especially in patients with high frailty. Periprocedural optimization of medical comorbidities could directly have a positive impact on reducing readmission burden. Careful selection of patients for periprocedural antimicrobial therapy and early recognition of infectious complications should be emphasized as patients with high frailty are commonly readmitted with sepsis, as evident in our study. Further prospective studies are necessary to evaluate the impact of such measures on reducing frailty-associated adverse outcomes.

Our study has numerous strengths and some limitations. An important strength of our study is the large study population, which has been derived from one of the largest, multi-ethnic and most inclusive inpatient databases in the US. With the weighted nature of the sample, the results of our study are generalizable and applicable to all index hospitalizations and readmissions in the US, offering therapeutic endoscopists real-world data. Despite these strengths, we acknowledge all the limitations associated with our study. The NRD database does not contain information on the etiology, hospital course, pharmacological data, and other treatment aspects of the disease. It also lacks data on patient selection, pre-procedural evaluation, intra-procedural details, specific procedural techniques utilized during the procedure, operator preferences, and pharmacological aspects of management before, during, or after the procedure. Furthermore, due to the retrospective nature of the study design, all biases associated with retrospective studies are applicable to our study. In performing a multivariate regression analysis while evaluating readmission risk, we were unable to adjust for the reason for readmission, due to the nature of the NIS database. Lastly, the NRD is an administrative database maintained through data collection organizations that use the ICD coding system to store inpatient data. Hence, the possibility of human coding errors cannot be excluded. However, despite these limitations, we believe that the large sample size and a comprehensive analysis technique help us better understand the clinical outcomes of 30-day readmissions of ERCP in the US.

In conclusion, ERCP readmissions are a significant healthcare burden that can lead to patient morbidity, mortality, and decreased quality of life. Frailty is a common co-existent factor in patients undergoing ERCP, and it is vital to recognize its key role in readmissions following ERCP. In this study, we noted that frailty not only increases readmissions but also inpatient mortality, LOS, and THC. Furthermore, females are disproportionately affected. HFRS can serve as a vital tool in predicting adverse outcomes, incorporating a multi-disciplinary approach in patient care and careful patient selection to help improve outcomes in patients undergoing ERCP. Additional large prospective studies are essential to assess the utility of incorporating HFRS into complex clinical decision making in an attempt to identify individuals at higher risk of adverse outcomes.

## Figures and Tables

**Table 1:** Hospitalization characteristics of admissions with HFRS < 5 receiving endoscopic retrograde cholangiopancreatography in the United States.

Hospital Frailty Risk Score < 5			
Hospitalization Characteristics of Admissions Receiving ERCP			
	Index Admissions	Readmissions	*p*-Value
Total Number	520,302	42,807 (8.2%)	
Index admissions discharged alive	519,253		
Mean Age (years) ± Standard Error	55.2 (55–55.34)	59.32 (59.05–59.59)	
Age Groups			
18–34	98,107 (18.8%)	4719 (11%)	<0.001
35–49	96,935 (18.6%)	6978 (16.3%)	<0.001
50–64	138,701 (26.6%)	12,908 (30.16%)	<0.001
65–79	132,542 (25.47%)	13,012 (30.4%)	<0.001
≥80	54,015 (10.38%)	5188 (12.12%)	<0.001
Gender			
Male	210,179 (40.4%)	21,170 (49.45%)	<0.001
Female	310,122 (59.6%)	21,637 (50.5%)	<0.001
Charlson Comorbidity Index (CCI)			
CCI = 0	257,170 (49.43%)	13,073 (30.54%)	<0.001
CCI = 1	117,029 (22.49%)	7935 (18.54%)	<0.001
CCI = 2	58,660 (11.27%)	6883 (16.08%)	<0.001
CCI ≥ 3	87,441 (16.81%)	14,915 (34.84%)	<0.001
Hospital Region			
Metropolitan (Large and small)	498,053 (95.72%%)	41,024 (95.83%)	<0.001
Micropolitan	18,795 (3.6%)	1363 (3.18%)	<0.001
Non-urban	3452 (0.6%)	420 (0.98%)	<0.001
Hospital Bed Size			
Small	75,109 (14.4%)	5381 (12.57%)	<0.001
Medium	134,156 (25.78%)	9747 (22.77%)	<0.001
Large	311,035 (59.78%)	27,627 (64.66%)	<0.001
Hospital Location and Teaching Status			
Metropolitan non-teaching	111,555 (21.44%)	7447 (17.4%)	<0.001
Metropolitan teaching	386,498 (74.28%)	33,577 (78.44%)	<0.001
Non-metropolitan	22,248 (4.28%)	1783 (4.17%)	<0.001

**Table 2:** Hospitalization characteristics of admissions with HFRS 5–15 receiving endoscopic retrograde cholangiopancreatography in the United States.

Hospital Frailty Risk Score 5–15			
Hospitalization Characteristics of Admissions Receiving ERCP			
	Index Admissions	Readmissions	*p*-Value
Total Number	343,781	43,309 (12.59%)	
Index admissions discharged alive	331,615		
Mean Age (years) ± Standard Error	68.3 (68.16–68.46)	67.53 (67.25–67.79)	
Age Groups			
18–34	14,831 (4.31%)	1515 (3.5%)	<0.001
35–49	27,290 (7.94%)	3706 (8.56%)	<0.001
50–64	77,566 (22.56%)	10,996 (25.39%)	<0.001
65–79	131,503 (38.25%)	17,049 (39.37%)	<0.001
≥80	92,586 (26.93%)	10,041 (23.19%)	<0.001
Gender			
Male	167,194 (48.63%)	22,031 (50.87%)	<0.001
Female	176,586 (51.37%)	21,277 (49.13%)	<0.001
Charlson Comorbidity Index (CCI)			
CCI = 0	61,753 (17.96%)	4951 (11.43%)	<0.001
CCI = 1	62,623 (18.22%)	5633 (13.01%)	<0.001
CCI = 2	56,299 (16.38%)	6650 (15.35%)	<0.001
CCI ≥ 3	163,104 (47.44%)	26,074 (60.21%)	<0.001
Hospital Region			
Metropolitan (Large and small)	332,440 (96.7%)	41,293 (95.35%)	<0.001
Micropolitan	9981 (2.9%)	1631 (3.7%)	<0.001
Non-urban	1359 (0.4%)	384 (0.89%)	<0.001
Hospital Bed Size			
Small	44,374 (12.9%)	5555 (12.8%)	<0.001
Medium	87,574 (25.47%)	10,486 (24.2%)	<0.001
Large	211,832 (61.62%)	27,266 (62.9%)	<0.001
Hospital Location and Teaching Status			
Metropolitan non-teaching	65,145 (18.95%)	7790 (18%)	<0.001
Metropolitan teaching	267,294 (77.75%)	33,503 (77.35%)	<0.001
Non-metropolitan	11,341 (3.3%)	2015 (4.65%)	<0.001

**Table 3:** Hospitalization characteristics of admissions with HFRS > 15 receiving endoscopic retrograde cholangiopancreatography in the United States.

Hospital Frailty Risk Score > 15			
Hospitalization Characteristics of Admissions Receiving ERCP			
	Index Admissions	Readmissions	*p*-Value
Total Number	21,332	2255 (10.57%)	
Index admissions discharged alive	19,559		
Mean Age (years) ± Standard Error	76.35 (76–76.6)	75.75 (74.9–76.5)	
Age Groups			
18–34	258 (1.21%)	10 (0.48%)	<0.001
35–49	546 (2.56%)	74 (3.32%)	<0.001
50–64	2610 (12.23%)	295 (13.1%)	<0.001
65–79	7470 (35.02%)	803 (35.62%)	<0.001
≥80	10,447 (48.97%)	1070 (47.48%)	<0.001
Gender			
Male	9730 (45.61%)	1005 (44.57%)	<0.001
Female	11,602 (54.39%)	1249 (55.43%)	<0.001
Charlson Comorbidity Index (CCI)			
CCI = 0	888 (4.17%)	58 (2.57%)	<0.001
CCI = 1	2770 (13%)	221 (9.8%)	<0.001
CCI = 2	3403 (15.8%)	317 (14.09%)	<0.001
CCI ≥ 3	14,269 (66.8%)	1657 (73.52%)	<0.001
Hospital Region			
Metropolitan (Large and small)	20,539 (96.28%)	2120 (94%)	<0.001
Micropolitan	679 (3.19%)	100 (4.48%)	<0.001
Non-urban	113 (0.53%)	34 (1.5%)	<0.001
Hospital Bed Size			
Small	2788 (%)	339 (%)	<0.001
Medium	5378 (%)	576 (%)	<0.001
Large	13,165 (%)	1339 (%)	<0.001
Hospital Location and Teaching Status			
Metropolitan non-teaching	4115 (19.29%)	454 (20.14%)	<0.001
Metropolitan teaching	16,423 (77%)	1665 (73.87%)	<0.001
Non-metropolitan	793 (3.72%)	134 (5.9%)	<0.001

**Table 4:** Indication for ERCP on index admission.

Indication for ERCP on Index Admission			
Indication	HFRS < 5	HFRS 5–15	HFRS > 15
Choledocholithiasis	71.80%	73.49%	72.64%
Biliary pancreatitis	28.11%	25.69%	19.59%
Cholangitis	0.00%	0.13%	1.21%
Malignant biliary obstruction	0.00%	0.00%	1%
Other	0.00%	0.50%	5.38%

**Table 5:** Readmission rates for admissions discharged alive after index admissions that received endoscopic retrograde cholangiopancreatography in the United States.

	HFRS < 5 (Referent)	HFRS 5–15	Unadjusted OR (95% CI)	*p*-Value	HFRS > 15	Unadjusted OR (95% CI)	*p*-Value
Readmission Rates in ERCP-Receiving Admissions Discharged Alive							
Overall Readmission Rate							
	42,583 (7.56%)	43,270 (11.17%)	1.67 (1.64–1.71)	<0.001	2477 (10.5%)	1.62 (1.52–1.73)	<0.001
Gender							
Male	21,076 (10.05%)	21,930 (13.68%)	1.41 (1.37–1.46)	<0.001	1159 (13.11%)	1.34 (1.23–1.47)	<0.001
Female	21,491 (6.9%)	21,320 (12.4%)	1.9 (1.84–1.96)	<0.001	1316 (12.28%)	1.87 (1.72–2.04)	<0.001
Age Groups							
18–34	4522 (4.6%)	1725 (11.79%)	2.76 (2.54–3.01)	<0.001	35 (13.7%)	3.29 (1.87–5.76)	<0.001
35–49	6839 (7.06%)	3848 (14.43%)	2.22 (2.08–2.36)	<0.001	75 (14.52%)	2.23 (1.55–3.22)	<0.001
50–64	12,966 (9.36%)	10,963 (14.63%)	1.65 (1.59–1.72)	<0.001	349 (15.37%)	1.75 (1.49–2.07)	<0.001
65–79	13,160 (9.96%)	16,652 (13.18%)	1.37 (1.32–1.42)	<0.001	977 (14.4%)	1.52 (1.37–1.68)	<0.001
≥80	5078 (9.44%)	10,061 (11.28%)	1.21 (1.15–1.28)	<0.001	1040 (10.68%)	1.14 (1.03–1.26)	0.009
Charlson Comorbidity Index (CCI)							
CCI = 0	12,865 (5%)	5424 (8.8%)	1.84 (1.75–1.94)	<0.001	80 (9.5%)	1.99 (1.38–2.87)	<0.001
CCI = 1	8168 (6.9%)	6360 (10.3%)	1.53 (1.45–1.61)	<0.001	269 (10.1%)	1.49 (1.25–1.79)	<0.001
CCI = 2	6989 (11.94%)	6679 (12.16%)	1.02 (0.96–1.07)	0.43	355 (11.01%)	0.91 (0.76–1.08)	0.303
CCI ≥ 3	14,544 (16%)	24,786 (16.1%)	0.95 (0.92–0.98)	0.007	1770 (13.8%)	0.79 (0.73–0.86)	<0.001
Hospital Bed Size							
Metropolitan non-teaching	5071 (6.765)	5008 (11.59%)	1.9 (1.82–1.99)	<0.001	297 (11.61%)	1.89 (1.65–2.17)	<0.001
Metropolitan teaching	10,233 (7.65)	10,351 (12.19%)	1.61 (1.57–1.65)	<0.001	605 (12.16%)	1.54 (1.43–1.66)	<0.001
Non-metropolitan	27,263 (8.75)	27,891 (13.7%)	1.78 (1.56–2.02)	<0.001	1572 (13.08%)	1.96 (1.34–2.86)	0.001

**Table 6:** Impact of frailty on readmission risks for admissions discharged alive after index admissions that received endoscopic retrograde cholangiopancreatography in the United States—adjusted odds after multivariate regression.

	HFRS < 5 (Referent)	HFRS 5–15	aOR (95% CI)	*p*-Value	HFRS > 15	aOR (95% CI)	*p*-Value
Readmission							
Overall							
	42,583 (7.56%)	43,270 (11.17%)	1.26 (1.23–1.29)	<0.001	2477 (10.5%)	1.12 (1.04–1.19)	0.001
Indication for ERCP							
Choledocholithiasis	42,547 (8.19%)	42,590 (12.99%)	1.26 (1.23–1.29)	<0.001	2134 (12.13%)	1.04 (0.97–1.12)	0.249
Biliary pancreatitis	11,241 (7.7%)	11,134 (13.06%)	1.51 (1.44–1.58)	<0.001	498 (13%)	1.37 (1.17–1.59)	<0.001
Other indication	9 (37.19%)	158 (15.66%)	0.28 (0.07–1.05)	0.061	78 (16.58%)	0.28 (0.074–1.08)	0.065
Gender							
Male	21,076 (10.05%)	21,930 (13.68%)	1.16 (1.12–1.20)	<0.001	1159 (13.11%)	1.02 (0.92–1.12)	0.683
Female	21,491 (6.9%)	21,320 (12.4%)	1.36 (1.31–1.41)	<0.001	1316 (12.28%)	1.11 (1.02–1.22)	0.017
Age Groups							
18–34	4522 (4.6%)	1725 (11.79%)	2.06 (1.87–2.27)	<0.001	35 (13.7%)	1.41 (0.74–2.7)	0.291
35–49	6839 (7.06%)	3848 (14.43%)	1.56 (1.45–1.67)	<0.001	75 (14.52%)	1.26 (0.84–1.87)	0.251
50–64	12,966 (9.36%)	10,963 (14.63%)	1.27 (1.21–1.33)	<0.001	349 (15.37%)	1.11 (0.93–1.33)	0.216
65–79	13,160 (9.96%)	16,652 (13.18%)	1.12 (1.08–1.17)	<0.001	977 (14.4%)	1.12 (1.00–1.25)	0.036
≥80	5078 (9.44%)	10,061 (11.28%)	1.11 (1.06–1.17)	<0.001	1040 (10.68%)	1.00 (0.90–1.11)	0.916
Charlson Comorbidity Index (CCI)							
CCI = 0	12,865 (5%)	5424 (8.8%)	1.69 (1.61–1.79)	<0.001	80 (9.5%)	1.79 (1.24–2.58)	0.002
CCI = 1	8168 (6.9%)	6360 (10.3%)	1.50 (1.42–1.58)	<0.001	269 (10.1%)	1.49 (1.23–1.79)	<0.001
CCI = 2	6989 (11.94%)	6679 (12.16%)	1.05 (0.99–1.11)	0.056	355 (11.01%)	0.97 (0.81–1.16)	0.8
CCI ≥ 3	14,544 (16%)	24,786 (16.1%)	1.01 (0.97–1.04)	0.539	1770 (13.8%)	0.91 (0.84–0.98)	0.026
Insurance							
Medicare	19,346 (10.08%)	28,400 (12.8%)	1.16 (1.12–1.19)	<0.001	2061 (12.4%)	1.08 (1.0–1.16)	0.041
Medicaid	6787 (7.6%)	4842 (14.76%)	1.41 (1.32–1.50)	<0.001	151 (14.09%)	1.05 (0.80–1.39)	0.693
Private insurance	13,408 (7%)	7997 (13.1%)	1.36 (1.29–1.44)	<0.001	203 (14.14%)	1.13 (0.9–1.42)	0.289
Self-pay	1727 (6.09%)	965 (12.8%)	1.68 (1.46–1.94)	<0.001	18 (12.69%)	1.13 (0.54–2.36)	0.737
Hospital Location and Teaching Status							
Metropolitan non-teaching	5071 (6.765)	5008 (11.59%)	1.32 (1.23–1.42)	<0.001	297 (11.61%)	1.10 (0.91–1.32)	0.303
Metropolitan teaching	10,233 (7.65)	10,351 (12.19%)	1.21 (1.15–1.28)	<0.001	605 (12.16%)	1.04 (0.90–1.21)	0.556
Non-metropolitan	27,263 (8.75)	27,891 (13.7%)	1.26 (1.22–1.30)	<0.001	1572 (13.08%)	1.07 (0.99–1.17)	0.083

**Table 7:** Mortality for admissions receiving endoscopic retrograde cholangiopancreatography in the United States.

Mortality in Admissions Receiving ERCP					
	Index Admissions	Readmissions	Odds Ratio	95% Confidence Interval	*p*-Value
Overall	14,693 (1.66%)	3907 (4.42%)	2.74	2.59–2.2.89	<0.001
Frailty Categories					
HFRS < 5	906 (0.17%)	507 (1.11%)	6.86	5.86–8.03	<0.001
HFRS 5–15	12,021 (3.49%)	3165 (7.3%)	2.17	2.04–2.31	<0.001
HFRS > 15	1764 (8.2%)	235 (10.44%)	1.29	1.04–1.60	<0.001

HFRS = hospital frailty risk score. Unadjusted odds ratios were obtained from univariate regression analysis.

**Table 8:** Healthcare burden for 30-day readmissions that received endoscopic retrograde cholangiopancreatography in the United States.

Healthcare Burden in Admissions Receiving ERCP							
	HFRS < 5 (Referent)	HFRS 5–15	Difference (95% CI)	*p*-Value	HFRS > 15	Difference	*p*-Value
THC *	68,034	118,996	50,962 (48,854–53,069)	<0.001	195,584	127,550 (120,581–134,519)	<0.001
LOS ^	4.22	8.49	4.26 (4.19–4.34)	<0.001	15.13	10.9 (10.52–11.28)	<0.001

* Total hospitalization charges in U.S. dollars, ^ length of stay in days.

**Table 9:** Commonly identified principal 30-day readmission diagnoses following ERCP, stratified by degree of frailty.

	HFRS < 5		HFRS 5–15		HFRS > 15	
	Readmission diagnosis	Proportion	Readmission diagnosis	Proportion	Readmission diagnosis	Proportion
1	Sepsis	7.90%	Sepsis	14.80%	Sepsis	22.22%
2	Pancreatic cancer	6.97%	Pancreatic cancer	4.69%	Acute kidney injury	4.16%
3	Acute pancreatitis	6.01%	Acute pancreatitis	2.82%	Aspiration pneumonia	2.79%
4	Choledocholithiasis	4.97%	Acute kidney injury	2.80%	Heart failure	2%
5	Unspecified postprocedural complications	2.31%	Choledocholithiasis	2.30%	Pancreatic cancer	1.17%
6	Cholangiocarcinoma	1.84%	Heart failure	1.70%	Cerebral infarction	1.08%
7	Cholangitis	1.54%	Cholangiocarcinoma	1.32%	Enterocolitis due to Clostridium difficile	0.44%

## Data Availability

Restrictions apply to the availability of these data. Data were obtained from Agency for Healthcare Research and Quality and are available from https://hcup-us.ahrq.gov/tech_assist/centdist.jsp (accessed on 1 February 2024) with the permission of the Agency for Healthcare Research and Quality.
